# Supplementary material for: Antigen-Specific T Cell Immunotherapy Targeting Claudin18.2 in Gastric Cancer
Source: Cancers (Basel). 2022 Jun 2;14(11):2758. doi: 10.3390/cancers14112758 (PMC9179507; doi:10.3390/cancers14112758)
Supplement: Supplementary file 1 [file cancers-14-02758-s001.zip › cancers-1730645-supplementary/Supplementary Figure.pdf]

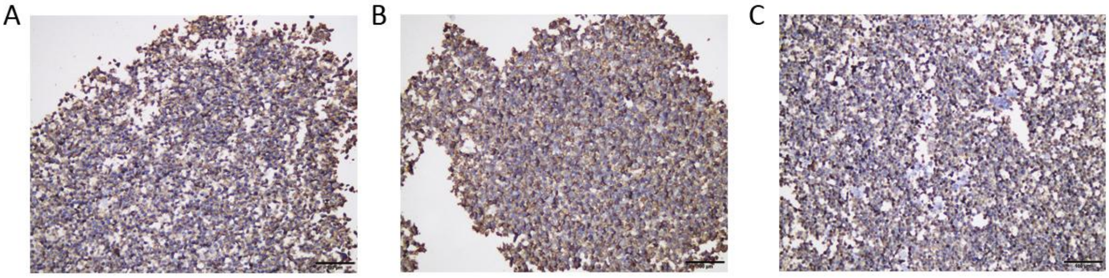

Figure S1 Claudin18.2 expression level in NUGC4 **(A),** AGS **(B)** and KE39 **(C).** All of the three GC cell lines were Claudin18.2 positive. The magnification was 200X.

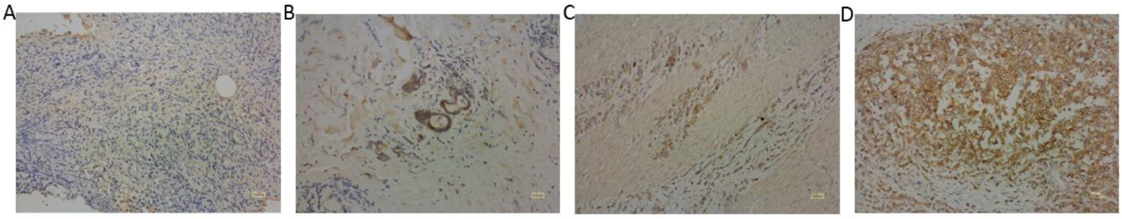

Figure S2 Immunohistochemical staining for Claudin18.2 in GC paraffin-embedded tissues. **A** 0, no membrane or cytoplasmic staining; **B** 1+, weak membrane or cytoplasmic staining; **C** 2+, moderate membrane or cytoplasmic staining; **D** 3+, strong membrane or cytoplasmic staining. The magnification was 200X.

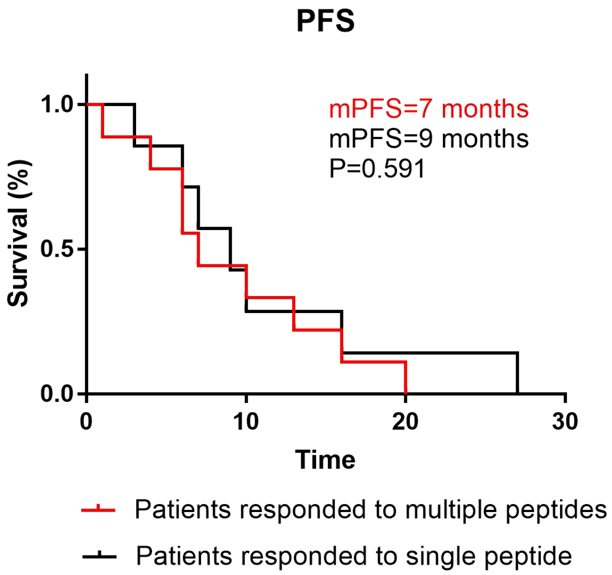

Figure S3 Relationship of multiple Claudin18.2 peptides reactivity and mPFS in gastric cancer patients. mPFS of multiple peptides reactive group was 7 months, mPFS of single peptide reactive group was 9 months, P<0.05 was considered statistically significant.

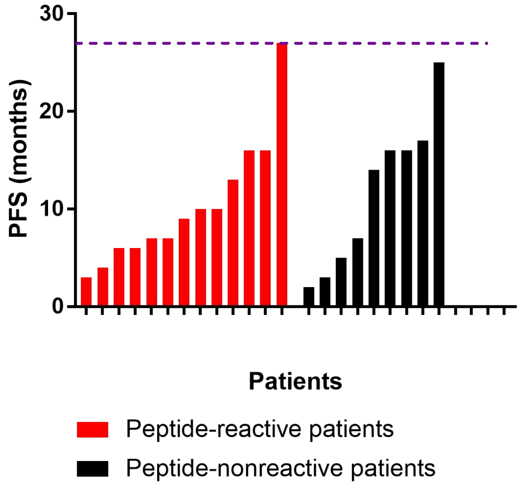

Figure S4 PFS data for each patients. The dotted line in purple represent for the longest PFS in all patients that were collected.
