# Supplementary material for: Antigen-Specific T Cell Immunotherapy Targeting Claudin18.2 in Gastric Cancer
Source: Cancers (Basel). 2022 Jun 2;14(11):2758. doi: 10.3390/cancers14112758 (PMC9179507; doi:10.3390/cancers14112758)
Supplement: Supplementary file 1 [file cancers-14-02758-s001.zip › cancers-1730645-supplementary/Supplementary Table.pdf]

Table S1. Characteristics of Claudin18.2 expression in patients with GC.

Factors		Total number	Positive	Negative	P-value	High	Low and Negative	P-value
Gender	Male	34	25	9	0.275	10	24	0.527
	Female	10	9	1		4	6	
Age	≥60	21	16	5	0.870	8	13	0.393
	<60	23	18	5		6	17	
TNM stage	I+II	6	5	1	0.146	1	5	0.692
	III	25	21	4		8	17	
	IV	11	6	5		4	7	
	Missing	2	2	0	-	1	1	-
Lauren classification	Intestinal type	11	9	2	0.792	5	6	0.201
	Diffuse type	14	12	2		3	11	
	Missing	19	16	3	-	6	13	-

'Missing' includes cases where classification was not applicable or not assessable; Positive, Claudin 18.2 positive; Negative, Claudin 18.2 negative; High, ≥40% of tumor tissues had specific Claudin18.2 staining with ⩾ 2+ intensity; Low and Negative, no Claudin18.2 expression or <40% of tumor tissues had specific Claudin18.2 staining.
